# Editorial: Complex Problem Solving Beyond the Psychometric Approach

**DOI:** 10.3389/fpsyg.2018.01224

**Published:** 2018-07-17

**Authors:** Wolfgang Schoppek, Annette Kluge, Magda Osman, Joachim Funke

**Affiliations:** ^1^University of Bayreuth, Bayreuth, Germany; ^2^Ruhr-Universität Bochum, Bochum, Germany; ^3^School of Biological and Chemical Sciences, Queen Mary University of London, London, United Kingdom; ^4^Universität Heidelberg, Heidelberg, Germany

**Keywords:** complex problem solving, dynamic decision making, system control, knowledge acquisition, thinking and reasoning

In 2005, Quesada et al. (2005) titled a paper “Complex problem-solving: A field in search of a definition.” Thirteen years later, it seems that the field has found it in the form of multiple (or minimal) complex systems such as MicroDYN. While MicroDYN certainly has brought the field a boost of attention and serves as a standard of comparison, not all researchers agree that it is an appropriate operationalization for complex problem solving (CPS). So is the field still searching? A pessimist would affirm this. However, the present collection of articles shows that the search is productive. For the intended scope of the research topic, please refer to the overview.

First, there is a number of conceptual papers. Dörner and Funke stake out the domain of complex problem solving. They claim that complex real-life problems need to be an important part of it. To bridge the gap between internal and ecological validity they recommend utilizing a broad range of research methods. Güss et al. make an argument for incorporating motivation into theoretical considerations about CPS. They substantiate their claim with the analysis of a thinking-aloud protocol of a subject (having worked on the WINFIRE simulation) with respect to three theories of motivation. Holt and Osman give an overview of various approaches to cognitive modeling of dynamic system control. They present strengths and weaknesses of those and conclude that due to the limitations of each single approach hybrid models are most promising. Huber presents theoretical considerations and reviews results about representation and evaluation in decision making. He had identified an advantages first principle, which is cushioned with the use of risk defusing operators. While this research has been conducted in the context of classical decision-making, it appears worthwhile to incorporate its principles into models of CPS. Overall, these papers give a good impression of the diversity of research questions and approaches within CPS and on its borders. Classifying these four papers as conceptual does not mean that the other papers are devoted to pure empiricism. Many of those contain elaborated forms of theoretical reasoning.

A next class of papers can be characterized by using a correlational methodology. Süß and Kretzschmar investigated the significance of intelligence and knowledge for performance in two different microworlds: a complex real-life oriented system (Tailorshop) that is largely in line with common knowledge, and a complex artificial world problem (FSYS) that was designed to minimize the influence of prior knowledge. The authors interpret their results as evidence that there is little reason to assume the existence of an ability construct CPS that explains variance in control performance over and above knowledge and reasoning. Molnár and Csapó present a large cross-sectional data set of strategy use in MicroDYN. They classified knowledge acquisition strategies with respect to effectiveness ex ante and found the expected developmental effects. The finding that using effective strategies, although being a predictor of control performance, is nonetheless neither necessary nor sufficient for high performance, shows that things are more intricate as desired, even in the rather plain environment of MicroDYN. In another large study with first year university students, Csapó and Molnár correlated several academic test scores, self-report measures of learning strategies, and MicroDYN performance. Besides the well-established relations among math scores, science scores, and control performance, the study revealed significant paths from elaborative (+) and memorizing (–) learning strategies to math and science test scores. Baars et al. present a study about the involvement of several affective and motivational variables on self-regulated learning of complex hereditary problems. Surprisingly, they found correlations mainly with negative variables: Negative affect, perceived mental effort and low self-assessment accuracy predicted low posttest problem-solving performance, whereas autonomous motivation was not a significant predictor. These results show that it is advisable to assist novice problem-solvers to regulate their mood when it comes to realistic self-assessment. The contribution of Hagemann and Kluge is the only one that addresses CPS in teams. This is an important aspect of solving problems in the real world. The requirement to coordinate team activities can be a source of additional complexity. In the context of a “model of the idealized teamwork process,” the authors investigated the hypothesized relations of cohesion, trust, and collective orientation and found that collective orientation had an effect on team performance mediated by the teams' coordination behavior.

In a third set of contributions, authors used experiments as their primary method. Beckmann et al. argue, based on a person task situation (PTS) framework, that it is important to distinguish difficulty from complexity. They demonstrate the implications of their claim with an experiment that varied semantic content of the system to be controlled and the assignment of tasks. The results confirmed the expected effects of complexity (induced by situation and task) on the observed difficulty. Schoppek and Fischer compared MicoDYN with a set of more dynamic, real-time driven control tasks (Dynamis2) in a transfer experiment. Besides the expected correlations among control performances in the two tasks and figural reasoning, the experiment revealed positive transfer from MicroDYN to Dynamis2, which was mediated by the use of a specific variant of the VOTAT strategy. Kumar and Dutt tested the effects of a dynamic climate change simulator (a stock-flow scenario) on misconceptions about CO_2_ accumulation in the atmosphere. They showed under various conditions that working with the dynamic climate change simulator reduced the misconceptions “correlation heuristic” and “violation of mass balance.” Prezenski et al. present an ACT-R model of a multidimensional classification task. The model combines an exemplar-based and a rule-based approach. It includes perceptual-motor and metacognitive aspects and is able to reproduce the essential effects of the underlying experiment.

At first sight, the diversity of research questions and methods in these contributions seems to be a severe hindrance to drawing conclusions. However, there are crosslinks that can give orientation in this maze.

1. Four papers agree in their appraisal that *motivational variables and processes* need to be considered in the investigation of CPS (Baars et al.; Dörner and Funke; Güss et al.; Hagemann and Kluge). This claim is as plausible as it is hard to prove: Studies have often failed to demonstrate pronounced effects of (global) motivational variables. Therefore, researchers need to incur the laborious approach of identifying and measuring proximal motivational variables that accompany the problem solving process.
2. Several papers address the role of *knowledge in CPS*. The considered knowledge types range from learning instances (e.g., Kumar and Dutt) to explicit knowledge about the structure of a system (e.g., Csapó and Molnár). The significance of prior knowledge is ambiguous: It can be helpful for successful problem solving (Süß and Kretzschmar), but also detrimental (Beckmann et al.). Although “knowledge” is one of the most elaborate topics within CPS, a model that unites several knowledge types in one framework is still a distant prospect (but see Schoppek and Fischer).
3. The question about *strategies for CPS* is closely related to the previous topic, because many of the strategies being investigated in the present collection of papers are serving the purpose of knowledge acquisition: VOTAT (Molnár and Csapó), VONAT (Beckmann et al.), and PULSE (Schoppek and Fischer). In addition, Huber's advantages first principle and Kumar and Dutt's correlation heuristic remind us of the possibility that in CPS not every course of action is so clearly scripted as in VOTAT.
4. As *cognitive models of CPS* need to specify knowledge and strategy, they embrace the previous two topics. Even though cognitive models have been used in that context for a while (e.g., Fum and Stocco, 2003), we believe that this methodology still helps to gain a better understanding of the interplay of strategies and the various knowledge types.
5. A final crosslink is constituted by the use of the minimal complex system MicroDYN (Csapó and Molnár; Molnár and Csapó; Schoppek and Fischer), showing that even though many authors take a skeptical view toward MicroDYN as the operationalization of CPS per se, it is still useful as a well-documented point of reference.

We hope that the present collection of articles will stimulate the exchange among researchers in the field of CPS, which is necessary to overcome potential separation. We also hope that it will serve as a guidepost on the way to an architecture of complex problem solving. Summarizing the present proposals, such a differentiated architecture would be hybrid and hierarchical, in order to incorporate diverse elements such as instance-based learning, rule induction, decision-making, and motivational variables.

## Author contributions

WS wrote the first draft of the editorial. All other authors commented on this draft and contributed improved or additional text.

### Conflict of interest statement

The authors declare that the research was conducted in the absence of any commercial or financial relationships that could be construed as a potential conflict of interest.
